# Comparing the effectiveness of behavioral activation in group vs. self-help format for reducing depression, repetitive thoughts, and enhancing performance of patients with major depressive disorder: a randomized clinical trial

**DOI:** 10.1186/s12888-024-05973-z

**Published:** 2024-07-19

**Authors:** Soleiman Saberi, Reza Ahmadi, Sahel Khakpoor, Reza Pirzeh, Mohsen Hasani, Latif Moradveisi, Omid Saed

**Affiliations:** 1 Department of Clinical Psychology, School of Medicine, Zanjan University of Medical Sciences, Zanjan, Iran; 2 Department of Clinical Psychology, School of Medicine, Shahid Beheshti University of Medical Sciences, Tehran, Iran; 3 Department of Clinical Psychology, School of Behavioural Sciences, University of Social Welfare and Rehabilitation Sciences, Tehran, Iran; 4 Department of Psychiatry, School of Medicine, Zanjan University of Medical Sciences, Zanjan, Iran; 5 Behavioral Disorders and Substance Abuse Research Center, Hamadan University of Medical Sciences, Hamadan, Iran

**Keywords:** Behavioral activation group therapy, Depression, Rumination, Students, Social and academic function

## Abstract

**Background:**

Behavioral activation has gained increasing attention as an effective treatment for depression. However, the effectiveness of Behavioral Activation Group Therapy (BAGT) in controlled conditions compared to its self-help programs requires more investigation. The present study aimed to compare their effectiveness on depressive symptoms, repetitive negative thinking (RNT), and performance in patients with major depressive disorder (MDD).

**Methods:**

In this randomized clinical trial, 40 patients diagnosed with Major Depressive Disorder (MDD) were recruited based on a structured clinical interview for DSM-5 (SCID-5). Participants were allocated to BAGT (*n* = 20) and self-help behavioral activation (SBA; *n* = 20) groups. BAGT received ten weekly sessions (90 min), while the SBA group followed the same protocol as the self-help intervention. Participants were evaluated at pre-treatment, post-treatment, and the 2-month follow-up using the Beck Depression Inventory-II (BDI-II), repetitive thinking questionnaire (RTQ-31), and work and social adjustment scale (WSAS).

**Results:**

The results of a Mixed ANOVA analysis revealed that participants who underwent BAGT showed significant improvement in depression, rumination, work, and social functioning post-treatment and at the 2-month follow-up. However, the SBA group did not show significant changes in any outcome. The study also found that, based on clinical significance, 68% of the BAGT participants were responsive to treatment, and 31% achieved a high final performance status at the 2-month follow-up.

**Discussion:**

BAGT was more effective than SBA in MDD patients. Participants’ engagement with self-help treatment is discussed.

**Trial registration:**

The present trial has been registered in the Iranian Registry of Clinical Trials Center (IRCT ID: IRCT20181128041782N1|| http://www.irct.ir/) (Registration Date: 04/03/2019).

## Introduction

Major depressive disorder (MDD) is characterized by low mood and loss of interest in activities. Additionally, patients with MDD face a range of mood, behavioural, cognitive, and physiological symptoms, leading to clinical distress and disrupted function [1]. MDD is one of the foremost reasons for disability and plays a significant role in the global disease burden [2]. The prevalence of MDD varies from 2 to 21% [3], while it is higher in student populations and was reported at 10–48% [4–6]. Due to the adverse and widespread consequences of MDD, the development of medications and psychotherapies has been considered, some of which have been ascertained to be effective, including medication [antidepressant; 7], psychoanalysis [8], interpersonal psychotherapy [9], and cognitive-behavioral therapy [CBT; 10]. Although there are differences between these therapies in feasibility, accessibility, and utility [11, 12], CBT has been the most popular in research and treatment.

Some limitations of CBT and the need for an effective evidence-based, short-term therapy resulted in component analysis of CBT, dividing it into main components, This analysis shows that the effectiveness of the behavioral component is independently comparable to the whole treatment [13]. This behavioral component is now recognized as a separate treatment with significant research evidence supporting its effectiveness. Behavioral activation (BA), compared with other therapies such as antidepressant medication [14], metacognitive therapy [15], mindfulness-based therapy [16], and acceptance and commitment therapy [17], did not show significant differences in the results. A clinical trial to comparing the effectiveness of BA and antidepressants patients undergoing BA treatment not only experienced greater symptom reduction than those on antidepressants but also found this treatment to be more effective, especially in individuals with severe depression [18]. Similarly, a meta-analysis demonstrated the effectiveness of BA group therapy, indicating that even when controlling for the severity of patients’ depression at baseline, the effects of this treatment remained significant [19].

Group therapy is as effective as individual therapy for various psychological disorders. Group therapy is more efficient because one therapist can reach many people at once, and it could be beneficial from a therapeutic and economic point of view [15]. Moreover, in group therapy, the stigma of psychological disorders can be decreased, and the functions of many people have improved at the same time [20].

In addition to clinical symptoms, depression has a detrimental impact on social, occupational, and recreational performance [21]. A study involving college students experiencing depressive symptoms revealed that depression affects their ability to derive pleasure from leisure activities, engage in social interactions, and participate in various activities [22]. Behavioral activation plays a crucial role in enhancing rewarding behaviors, thereby improving the psychosocial functioning of individuals with depression [23–25]. Another study conducted with first-year university students found that involvement in meaningful activities can enhance their social adjustment and augment the effectiveness of behavioral activation in alleviating depression symptoms [26].

Repetitive thought refers to recurrent thinking, such as rumination and worry, which can have both constructive and unconstructive effects. The negative aspect of repetitive thought is recognized as a transdiagnostic factor contributing to the development of various emotional disorders, and it plays a crucial role in their severity and persistence [27]. Consequently, addressing repetitive negative thinking (RNT) becomes a significant focus in cognitive-behavioral treatments for anxiety and depression [28]. A meta-analysis has indicated that cognitive-behavioral therapy has a notable impact on reducing RNT among depressed patients, particularly in interventions targeting negative repetitive thoughts [29]. While rumination-focused therapy has been identified as a tailored treatment for addressing RNT [30, 31], there is also evidence suggesting that behavioral activation therapy can effectively mitigate these thought processes [32, 33]. A study evaluating the effectiveness of a brief behavioral activation protocol for depressed adolescents demonstrated a significant reduction in depression and rumination following four treatment sessions [34]. This suggests that rumination is a modifiable target for behavioral activation therapy.

Although the available evidence regarding the efficacy of BAGT is promising, some researchers have suggested that self-help programs could be effective intervention choices for depressive patients [35, 36]. If the efficacy of self-help treatment programs is comparable to group clinical interventions, self-help programs could serve as affordable and easily accessible alternatives [37]. Nonetheless, concerns such as weak motivation to pursue treatment, feeling overwhelmed by program assignments, and difficulty in maintaining changes are recognized as significant obstacles in the path of self-help programs [37–39].

The present study aims to compare the effectiveness of the group format of behavioral activation with self-help in reducing depressive symptoms and repetitive thoughts and improving performance in students with MDD.

## Method

This two-armed randomized clinical trial (including BAGT and SBA condition) was conducted under the supervision and approval of the Iranian Clinical Trials Registry (ID: Blinded for review). In addition, it was conducted according to the CONSORT guidelines [40]. Screening of the participants was accomplished in two stages; in the primary screening, they were evaluated using BDI-II, then, based on the cut-pint of this inventory (BDI-II ≤ 14), people who were in the clinical range of depression were invited for secondary evaluation. At this stage, the participants were evaluated for clinical interview and evaluation of inclusion and exclusion criteria using a Structured Clinical Interview for DSM-5 (SCID-5). Participants were measured in terms of severity of depression, RNT, and performance in three stages pre-treatment, post-treatment, and two months follow-up.

### Sample size

Using G*power, the sample size for this study was determined to be 34. Considering that the current study was conducted on a small scale, the inputs of the function were considered in such a way that it is compatible with the conditions of the study. Based on that, the effect size (*d*) was 0.90, the error probability (*α*) was 0.05, and the power (*1 - β err prob*) was 0.70. The output of the analysis estimated the critical value of *t* to be 2.03 and the actual power to be 0.72. To reduce the possible effect of sample attrition on study power, 6 more participants than estimated were included in the study. As a result, the final sample size of the research was estimated to be 40 participants.

### Participants

At first, recruitment announcements for a study on depression were posted on the social media of universities in Zanjan, Iran. The trial was held in the Psychiatry Clinic of Zanjan University of Medical Sciences. Students who were interested in participating contacted the research evaluation team to participate in the primary screening by online BDI-II. Among those who participated in the first screening (*N* = 350), 92 students met the criteria to participate in the second evaluation (BDI-II ≥ 14). Finally, 40 participants were chosen by the inclusion and exclusion criteria. The inclusion criteria included principal diagnosis of MDD (based on SCID-5). Exclusion criteria also included (a) a history of psychosis, (b) substance abuse disorders, (c) bipolar mood disorder, (d) personality disorders, (e) depression related to a medical condition, (f) a history of psychological interventions from 2 years ago (more than five sessions), (g) using the psychotic medication, (h) history of hospitalization for depression. Furthermore, if the participants were absent for more than two sessions during the interventions or did not participate in any of the post-treatment and follow-up assessments, they were excluded from the study. The BAGT (*n* = 18) included 61.1% females (M = 21.67 years; SD = 2.33), and the SBA (*n* = 18) consisted of 72.2% females whose average ages were 22.67 years (SD = 2.14).

### Procedure

**Fig. 1:**
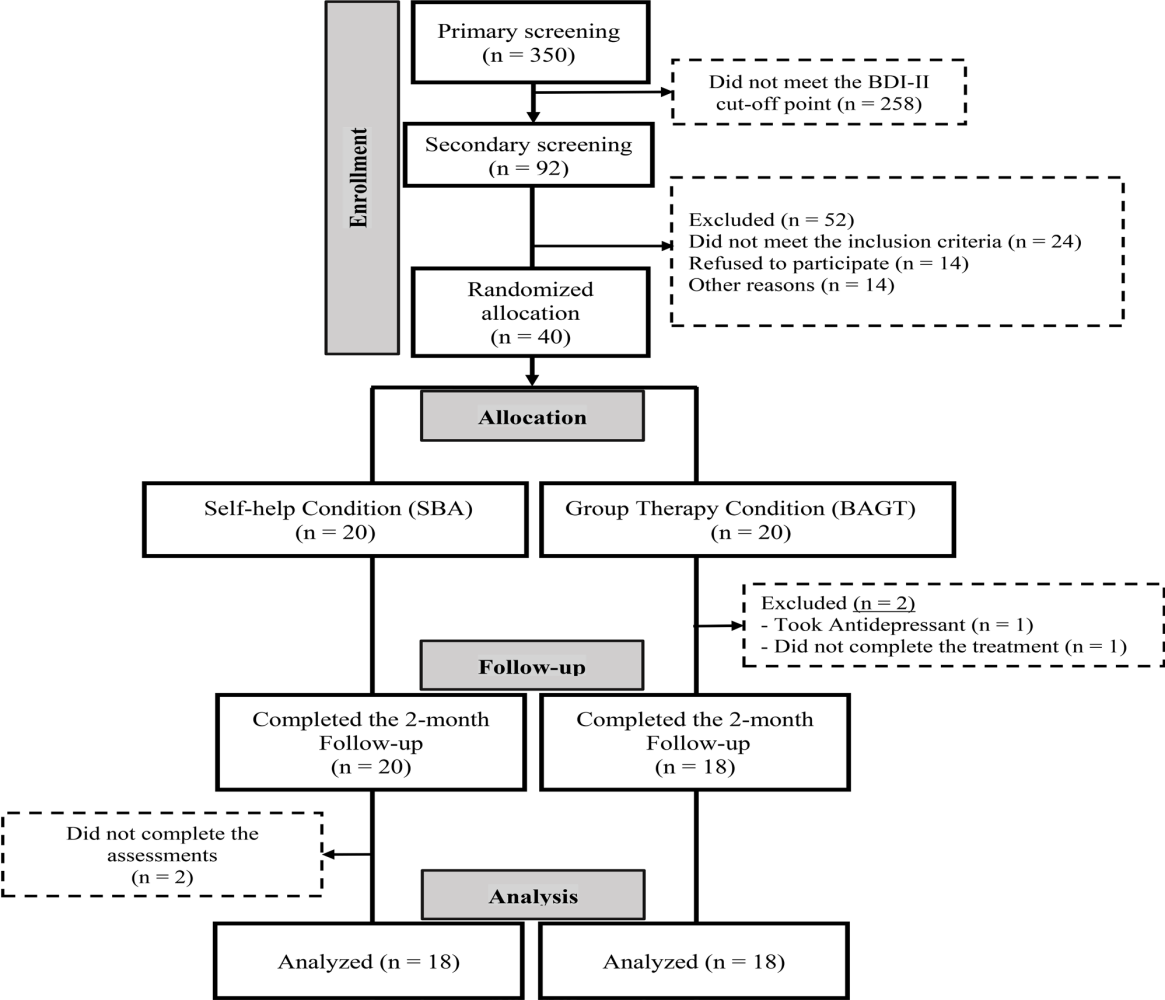
The study CONSORT flow diagram

Following the selection process outlined in the previous section, participants were required to complete an informed consent form. Out of the initial pool, 40 individuals were deemed eligible and were randomly assigned to the BAGT (*N* = 20) and the SBA (*N* = 20) conditions. According to the literature, the optimal group size for psychotherapy is 8 to 12 patients [41]. Therefore, participants in the BAGT study were randomly divided into two completely identical parallel groups. The self-help group received a booklet that was prepared and arranged according to the same protocol as BAGT, however, this group did not have the guidance of a therapist (further details provided in heading 2.4 Intervention). Participants in the SBA group were instructed to record the number of hours they intentionally spent engaged in reading and completing the booklet exercises on a weekly basis. This information was then reported to the group facilitator in order to facilitate an objective assessment of their level of involvement with the SBA program. During the study, two participants left the group therapy, and two others from SBA were excluded from the study (did not completing the assessments). Ultimately, the data of 36 participants were analyzed (Fig. 1).

### Randomization and blinding

The psychiatrist who conducted the clinical interview was blinded to the research goals. Meanwhile, the patients were not informed of their assigned group or of the activities in the other group during treatment. In the final stage the data were coded and provided to the data analyst. It is worth mentioning that a trained research assistant was responsible for allocation (using Random Number Generator 3.1), evaluations (pre-, post-, and follow-up), and data coding (prior to analysis).

### Treatment

BAGT condition engaged the participant in 10 interactive sessions with practical assignments, while participants in SBA group received a BA booklet. The participants were informed about the consideration to inform about any psychological and pharmacological treatment at the same time as the sessions. The group therapist was a trained clinical psychologist in behavioural activation for one year and had implemented the protocol many times in the clinical environment. All treatment sessions were conducted under an assistant professor of clinical psychology. In addition, the supervisor reviewed the recording of treatment sessions to assess the therapist’s adherence to the treatment protocol. Furthermore, SBA was selected as an accessible and cost-effective format of behavioural activation for comparison. In this study, SBA utilized a self-help booklet available online to participants.

### Group and self-help format

The present study used the revised version of behavioural activation program [42]. In this protocol, participants initially became aware of their state of life and depressive behaviours by monitoring activities. The BA model for depression was developed by information that collected through interview and activity monitoring forms, which were then shared with patients. In the next step, behaviour changes were started by targeting depressing activities and assessed using behaviour checklist form. In addition, in the final part of the treatment, the values ​​were clarified, each value area was divided into short-term and long-term behavioural goals, and these value-oriented activities were written and monitored in the behavior checkout form. During the study, patients received ten treatment sessions; the first four sessions were twice a week, and the rest were held once a week.

The SBA group received the same protocol in the form of self-help. In other words, the content of the protocol and the arrangement of the protocol presentation (amount of content presented in each session, order of content presentation, interval between sessions, and answering possible questions) were similar. The therapist’s role in this group was to send the material to the participant in a structured way and answer questions if there were any.

### Outcomes

#### Structured clinical interview for DSM-5-clinician version (SCID-5-CV)

This interview [43] helps the clinician to examine the patient’s diagnostic status according to the criteria of the diagnostic and statistical manual of mental disorders- fifth edition (DSM-5). This version has a high level of clinical diagnosis and interview agreement (between 70 and 90%), and its sensitivity and kappa’s level for most diagnoses are estimated to be more than 70% [44]. Examining the characteristics of the Persian version of SCID-5-CV indicates that the Kappa level (not including anxiety disorders) and the sensitivity of this test is more than 80%, which is good and reliable [45].

#### Beck depression inventory-II (BDI-II)

BDI-II has included 21 items designed to evaluate the severity of depressive symptoms. The scores of this questionnaire are in the range of 0 to 63, which are categorized into four categories: *lack of depression symptoms* (0 to 13), *mild depression* (14 to 19), *moderate depression* (20 to 28), and *severe depression* (28 to 63) [46]. This widely used questionnaire has good internal consistency (α = 0.83 to 0.93) in various populations [46–48]. Y-P Wang and C Gorenstein [48] also reported good test-retest reliability (0.73 to 0.96) for it. In the BDI-II Persian version study, the internal consistency of the BDI-II was reported as 0.87, and the post-test reliability was 0.74 [49].

#### Repetitive thinking questionnaire-31 (RTQ-31)

This self-report questionnaire involves 31 items designed by PM McEvoy, AE Mahoney and ML Moulds [50] to measure perseverative thinking. It consists *repetitive negative thinking (RNT)* subscale and *absence of repetitive thinking (ART)*. A 5-point Likert scale ranging from *not true* to *very true* is used to score items. The evidence indicates good internal consistency for the RNT subscale (α = 0.89), while the subscale of the absence of repetitive thinking (α = 0.62) is not acceptable. Also, this scale’s overall Cronbach’s alpha is reported as 0.94 in an Iranian sample [51].

#### Work and social adjustment scale (WSAS)

It was used to assess the following dysfunction impairment domains: (1) education, (2) home management, (3) social leisure activities, (4) private leisure activities, and (5) relationships with others. This self-report instrument measures the level of impairment with 5 items scored between 0 (indicating no impairment) and 8 (indicating severe impairment) and the score range of it is 0–40 (greater than 20 indicate severe psychopathology and symptomology). The patients were instructed to complete the items concerning their current depression [50]. In the studies conducted in Iran by Soleimani et al. and Nikandish et al., it has been found that it had a significant correlation with depression (*r* = .66, *p* < .001) and its test-retest reliability was 0.88 and 0.69 [52, 53] In the present study, the participants were student, so education is considered equal to the work and its items have been changed from work to education.

### Statistical strategy

The quality of the data collection for this study and the way of coding them were done under the direct supervision of the director of the clinical psychology department of Zanjan University of Medical Sciences. The method of data collection in each of the three stages of evaluation was reported to them. After collecting and entering data in SPSS 22 software, the data analyst provided the information in encrypted form. First, the Chi-square test compared group differences in demographic variables. In addition, according to the assumption of normal distribution of variables based on the Kolmogorov-Smirnov (K-S) test and equality of variances in groups based on Levine’s test, the independent t-test was used for evaluating baseline clinical differences of the outcomes (BDI, RTQ, and WSAS). Furthermore, a mixed model ANOVA was conducted to investigate the main and interaction effects during the study. The partial eta squared was used to examine the changes’ effect size. In situations where there was missing data, such as when participants missed more than three intervention sessions or dropped out before post-treatment and follow-up assessments, intention-to-treat (ITT) analysis was used to estimate the potential effect. Researchers who are interested in knowing how to collect, code, secure, and store the data of this study can obtain the required information by contacting the corresponding author of this study.

## Results

### Baseline differences

Examining the baseline demographic differences between groups showed no significant difference regarding gender and age (Table 1). Likewise, differences between groups in the pre-treatment of outcome variables were not significant (*p* > .05). It means the baseline differences in BDI (*t* = 0.313, *p* = .756), RTQ (*t* = 0.158, *p* = .875) and WSAS (*t* = -0.136, *p* = .892) was controlled.

**Table 1:** Baseline Differences Regarding Demographic Characteristics

Variables		BAGT	SBA	Total	χ^2^	*P*-value
		*N* (%)	*N* (%)	*N* (%)		
Gender	Male	7 (38.9)	5 (27.8)	12 (3.33)	0.500	0.480
	Female	11 (61.1)	13 (72.2)	24 (66.7)		
	Total	18 (100)	18 (100)	36 (100)		
Marital Status	Single	20 (100)	20 (100)	40 (100)		
Education Level	Undergraduate Student	13 (65)	12 (60)	25 (62.5)	0.107	0.744
	Postgraduate Student (M.Sc., M.D.)	7 (35)	8 (40)	15 (37.5)		
Depression Severity	Mild	9 (45)	11 (55)	20 (50)	1.20	0.549
	Moderate	8 (40)	8 (40)	16 (40)		
	Severe	3 (15)	1 (5)	4 (10)		
		Mean ± SD	Mean ± SD	Mean ± SD	t	P-value
Age		21.76 ± 2.33	22.67 ± 2.14	22.17 ± 2.26	34.1	0.189

Note BAGT: Behavioral Activation Group Therapy; SBA: Self-help Behavioral Activation

### Engament of SBA group

To evaluate how engaged the participants in the SBA group were with the self-help treatment content, we requested that they track the time they spent reading and doing the exercises in the booklet. Analysis of the data that was reported revealed a decline in the participants’ engagement over 8 weeks (see Table 2).

**Table 2:** The degree of involvement of SBA group by hours

	*N*	Range	Min	Max	Sum	Mean	Std. Deviation
Week 1^*^	18	11	1	12	121	6.7	3
Week 2^*^	18	11	1	12	89	5	3.1
Week 3	18	10	0	10	49	2.7	2.5
Week 4	18	6	0	6	43	2.4	1.5
Week 5	18	5	0	5	42	2.3	1.3
Week 6	18	5	0	5	37	2	1.2
Week 7	18	3	0	3	22	1.22	0.80
Week 8	18	4	0	4	19	1.06	1.3

Note In the first two weeks, similar to the BAGT group, the contents of the sessions were provided to the participants twice

### Effectiveness of treatment

The K-S test indicated that the distribution of dependent variables, including BDI, RTQ, and WSAS, was normal (*P* > .05). In addition, Levene’s test showed that the assumption of homogeneity of variances is confirmed in all variables (*P* > .05).

For comparing BDI-II, RTQ-31, and WSAS in two conditions (BAGT and SBA) and three-time points (pre-treatment, post-treatment, and follow-up), mixed ANOVA was used. The sphericity assumption was confirmed for BDI (*Mauchly’s W* = 0.694, *P* > .05) and WSAS (*Mauchly’s W* = 0.702, *P* > .05) in the SBA; it was not observed for BDI, RTQ-31, and WSAS in the BAGT and RTQ-31 in the SBA. Therefore, the Huynh-Feldt correction was applied due to the violation of sphericity for these variables. Mixed ANOVA revealed the significant main effect of time for the BDI, RTQ-31 (*P* < .001), and WSAS (*P* < .01), which shows outcome variables scores in the BAGT had changed significantly from pre-treatment to 2-month follow-up. Nevertheless, it was indicated that there is no significant main effect of time for the SBA (*P* > .01). Furthermore, between-group comparisons indicated that the main effect of the group was significant for BDI (*P* < .001) and WSAS (*P* < .01), while it was not for RTQ-31. The scores of BDI (*P* < .001) and WSAS (*P* < .01) in BAGT significantly changed compared to the SBA, while the main effect of the group was not significant for RTQ-31. In other words, the BDI-II and WSAS were significantly reduced in the BAGT compared to the SBA. Moreover, the interaction effect of the time × group was significant, which shows a significant difference between BDI-II (*P* < .001), RTQ-31 (*P* < .01), and WSAS (*P* < .01) from pre-treatment to 2-month follow-up. Finally, the Eta test showed that BAGT had reduced BDI-II, RTQ-31, and WSAS by 58.2, 55.6, and 45.6%, respectively (Table 2).

### Maintenance of treatment outcomes

**Fig. 2:**
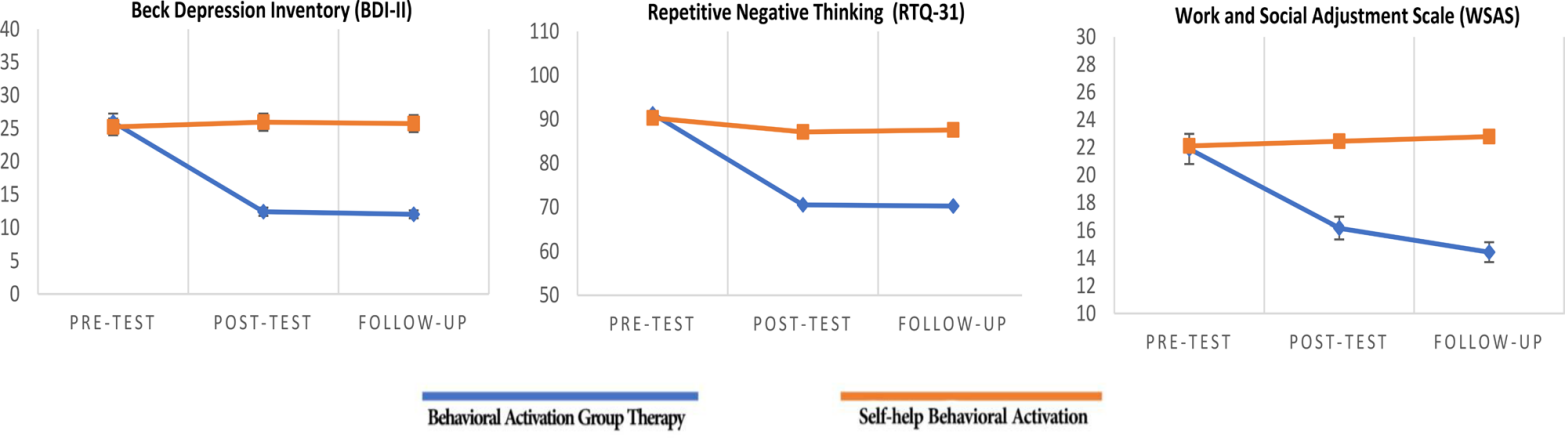
Comparison of outcomes in pre-treatment, post-treatment, 2-month follow-up

A significant decrease in BDI-II, RTQ-31, and an increase in WSAS from pre-treatment to post-treatment and follow-up was shown by the least significant difference test (LSD) in the BAGT (*P* < .01). Furthermore, significant improvement in WSAS scores was observed at the follow-up stage (*p* < .01), while there was no significant difference in BDI-II and RTQ-31 scores from post-treatment to follow-up (*P* > .05) (Fig. 2). As a result, it seemed that the patients had not only saved the changes but had gained more achievements in terms of performance.

### Clinical significance

An algorithm introduced in previous studies was used to evaluate the changes and achievements of the participants from the clinical perspective [54, 55].In this algorithm, two states refer to clinical significance, treatment responder status and high end-state functioning (HESF), which were considered based on participants’ post-treatment, post-test, and 2-month follow-up stages. This study indicated a 30% decrease in the BDI-II and a 30% increase in the WSAS scores as a response to treatment. Moreover, a 60% decrease in the BDI scores and a 60% increase in the WSAS were also indicated as HESF. The analyses indicated that the BAGT led to significant clinical changes in the post-treatment and 2-month follow-up (Table 3) and Table 4.

**Table 3:** Outcome measures at each Assessment

	BAGT		SBA		t (df)	F (df)	F (df)	F (df)	Eta
	M	SD	M	SD	Pre-treatment comparison	Within-group comparison	Between-group comparison	Interaction time×;group	
BDI-II									
Pre-treatment	25.94	7.04	25.22	6.80	*t*(34) = 0.313, *p* = .756	*F*(2,33) = 12.51, *p* < .001	*F*(1,34) = 21.54, *p* < .001	*F*(2,33) = 13.58, *p* < .001	0.582
Post-treatment	12.39	7.50	25.94	7.02					
Follow-up	12.00	6.31	25.72	7.80					
RTQ-31									
Pre-treatment	91.22	18.74	90.28	16.98	*t*(34) = 0.158, *p* = .875	*F*(2,33) = 12.52, *p* < .001	*F*(1,34) = 3.88, *p* > .05	*F*(2,33) = 7.10, *p* < .01	0.556
Post-treatment	70.50	19.65	87.11	16.80					
Follow-up	70.22	19.16	87.56	17.26					
WSAS									
Pre-treatment	21.89	4.32	22.11	5.40	*t*(34)=- 0.136, *p* = .892	*F*(2.33) = 5.48, *p* < .01	*F*(1,34) = 7.39, *p* < .01	*F*(2.33) = 7.87, *p* < .01	0.456
Post-treatment	16.17	6.80	22.44	5.79					
Follow-up	14.44	7.52	22.78	6.55					

BAGT Behavioral Activation Group Therapy; SBA: Self-help Behavioral Activation; *p* < .05, *p* < .01, *p* < .001

**Table 4:** Percentage of improvement in two states of treatment responders and high end-state functioning

Variables	Post-treatment				Follow-up			
	N	Treatment responders (%)	N	High end-state functioning (%)	N	Treatment responders (%)	N	High end-state functioning (%)
BDI-II, WSAS	10	62.5	1	6.2	11	68.75	5	31.25

## Discussion

BAGT led to significant changes in depression, RNT, and performance in students with MDD, while SBA did not cause significant changes. Participants in BAGT not only achieved significant changes post-treatment, but they continued to improve at the 2-month follow-up. The analysis of the SBA group participants’ time spent engaging with the self-help treatment content revealed that, despite making a strong effort in the initial weeks to review the booklet and complete its exercises, their involvement gradually decreased as the treatment progressed. However, the comparison of BAGT with SBA revealed significant difference in the changes in depression, RNT, and performance. Examining the *status of treatment responders* at post-treatment and follow-up indicated that more than half of the BAGT participants responded to treatment. However, the final performance to treatment was initially low at post-treatment but increased at follow-up so one-third of the participants showing improvement. Despite some previous studies suggesting that self-help conditions can be as effective as intervention groups [35, 56, 57], this study did not find a significant difference in depression, RNT and performance improvement of participants after self-help intervention.

No action was taken regarding the individual follow-up of the participants in the SBA group; Consequently, the lack of improvement in depressive symptoms is likely attributed to the participants’ disengagement with the treatment protocol and their failure to perceive the necessity of consistently completing assigned tasks. They probably reverted to past patterns of depression, hindering their activation.

Considering that clients were not compelled to do assignments—rather, they were offered two options: receiving therapeutic content and asking questions about the therapy or checking assignments—it can be inferred that providing additional content for depressed patients without external coercion does not significantly impact depression improvement.

It is suggested to employ the following methods to increase the engagement of the participants in the self-help programs: (a) follow up during the treatment by calling them [35], and (b) using mobile applications to send reminder and monitor the time and quality of participants’ engagement with content and assignments [58]. Also, in the researches it is suggested to count the number of homework sheets they have completed to assess the amount of engagement in the treatment [35].

Considering that the present study was conducted with the aim of comparing two group formats of BA in participants with MDD, the evidence should be interpreted clinically. Previous evidence has also supported that the group format as going out of home, being in a group, and social interaction itself are considered an active and anti-depressant behavior [59]. It seems that the potential of group therapy helps to improve the motivation of the participants at the beginning of the treatment, and in the continuation of the co-learning and empathy of the participants, it becomes effective in the continuation of making therapeutic changes [60]. For example, during the treatment, when the participants had achieved small changes in their daily lives, they received positive feedback and encouragement from other group members as their motivation to continue the changes. However, participants in the SBA group who received self-help interventions via Computer or smartphones faced several barriers that made it difficult for them to engage with the treatment content. For instance, they had to devote a long time to studying different parts of the self-help booklet, which is considered a heavy cognitive task for depressed patients. Additionally, considering that mobile phones are often associated with behaviors that exacerbate depression, such as aimlessly surfing social media [61], it was challenging for participants to stay focused on the booklet [62–65]. Furthermore, participants in the SBA group did not benefit from the advantages of the BAGT group and the active presence of the therapist. Based on this evidence, it can be concluded that while some benefits of self-help treatment, such as ease of access to treatment content, may not significantly improve effectiveness for patients with MDD in this treatment format, it instead necessitates more therapeutic elements.

Regarding the content of behavioural activation treatment, participants in activity-monitoring interventions, participants identified their depressing behaviours and gained insight into with how these behaviours hindered the attainment of positive reinforcement [42, 66]. Additionally, delineating life domains and values helped students commit to implementing short-term and long-term behavioural planning. This aligns with previous clinical trials demonstrating the effectiveness of BA for depressed adult patients [e.g., 18, 19]. Theoretically, addresses perpetuating factors, aversive control, and barriers preventing patients from accessing positive reinforcement [67]. Moreover, by increasing the frequency of adaptive activities, this treatment enhances patients’ sense of control and mastery of patients and leads to the experience of positive emotions [68, 69].

Although RNT are not well known in the context of BA, the present study suggests that this treatment can relatively reduce rumination. appears that the reduction of RNT in BA is simultaneously caused by some modules of this treatment and their impact on treatment outcomes. For instance, Monitoring daily activities and having a list of profitable activities to do can change the mentality of students from a mental state to an objective state, so they devote less time to rumination, specially engaging in activities that is valuable for them [70]. Additionally, engaging in productive activities and experiencing positive reinforcement during treatment can enhance patients’ mastery and self-efficacy [71]. In line with this, ER Watkins, CB Baeyens and R Read [72] argued that maintaining a clear and practical perspective can help reduce rumination and depression. The analysis of behavioural activation therapy outcomes indicated that engaging in daily activities can increase mood and feelings of pleasure [73], and even engaging in a video game intervention lead cognitive improvement and reduced rumination [74].

There is some research that have used WSAS as a part of assessments and it’s obvious that BA is effective in reducing the function impairment related work [52, 53] but there is little evidence regarding the effectiveness of BAGT on academic performance. The participants in the present study significantly improved their academic performance. BA therapy is task-oriented, objective, and transparent, which has caused it to extend the effects on the patient’s real-life environment while having relatively immediate impacts [68, 70]. Another factor contributing to the improvement in patients’ performance is the emphasis of behavioural activation therapy on skill acquisition. Through this approach,, the extinct behaviours reactivated again [75]. Furthermore, the group format of the protocol and active participation in group activities enhance social and communication skills, which are directly contributing to the improvement of patients’ academic-social adaptation [76]. Given that the present study was conducted in a community of students experiencing MDD, enhancing academic and social performance was one of the critical treatment objectives. Throughout the intervention, participants clarified their values and goals and engaging in functional activities related to education and social relationships. Utilizing problem-solving skills and actively participating in the group activities resulted in receiving positive feedback and reinforcements, encouraging the continuation of these behaviors.

## Conclusion

When MDD is addressed and treated, patients can discover more opportunities to enhance their mental health and develop their academic and professional skills. BA significantly benefits them, especially in a group format. Firstly, instead of engaging in mental analysis, they approach depression objectively. Secondly, they gain insight into the essence and prevalence of depression in academic settings and how other students react to depression. Thirdly, they provide feedback about each other’s behavioural barriers and changes during group interaction, something participants in self-help groups were deprived of; This highlights the importance of group interactions during treatment and overcoming treatment barriers, including low motivation in individuals with low mood. BAGT is effective for both reducing the clinical symptoms of depression and improving the performance of students making it suitable for practical implementation in academic settings.

This study encountered limitations that need to be considered in interpreting the results. The utilization of a small sample of students with MDD diminishes generalizability, hence future studies should explore the effectiveness of BA group therapy with larger and more diverse samples. Furthermore, it is essential to investigate the long-term consequences of treatment for reliability; Therefore, future studies contribute to the research literature by examining how participants respond to symptom relapse and functional decline after treatment. In addition, the present study did not accomplish the analysis of treatment components. Therefore, it is suggested that researchers examine the effect of each treatment component on the dependent variables. Moreover, in this study, the comparison group was assigned to SBA. It appears that the SBA yielded less clinical change compared to the BAGT. Nevertheless, it is plausible that utilizing booklets as a supplementary treatment or as a preventive measure for recurrence may prove to be effective. Hence, it is recommended that prospective studies explore SBA in individuals with milder or subthreshold depression, or as a method of preventing relapse. It is also recommended that the content of self-help manuals of BA be updated to increase their effectiveness. This can be achieved by presenting the booklets with audio and video content to prevent monotony. Additionally, providing self-help treatments through a dedicated mobile application can offer a structured approach to training and assignments. This can enable patients to progress step-by-step and receive a comprehensive evaluation of their development. Finally, online groups and forums that allow patients to ask questions in the presence of therapists can improve the outcomes of self-help treatments.

## Data Availability

The data are available from the corresponding author upon reasonable request and with the permission of Zanjan University of Medical Sciences.

## Abbreviations

BAGT: Behavioural Activation Group Therapy
SBA: Self-help Behavioural Activation
MDD: Major Depressive Disorder
CBT: Cognitive-Behavioral Therapy
BA: Behavioral Activation
DSM-5: Diagnostic and Statistical Manual of Mental Disorders, Fifth Edition
SCID-5-CV: Structured Clinical Interview for DSM-5-Clinician Version
SCID-5: Structured Clinical Interview for DSM-5
BDI-II: Beck Depression Inventory-II
RTQ-31: Repetitive Thinking Questionnaire-31
RNT: Repetitive Negative Thinking
ART: Absence of Repetitive Thinking
WSAS: Work and Social Adjustment Scale
